# Vitamin D Status and Its Determinants in Mexican Pregnant Women from a Rural and an Urban Area: A Comparative Study

**DOI:** 10.3390/ijerph18094571

**Published:** 2021-04-26

**Authors:** Mayra Chávez-Courtois, Estela Godínez-Martínez, Cinthya Muñoz-Manrique, Viviana Negrete-Martínez, Carla Patricia González-Leyva, Maricruz Tolentino-Dolores, Blanca Suárez-Rico, Guadalupe Estrada-Gutierrez, Otilia Perichart-Perera

**Affiliations:** 1 Nutrition and Bioprogramming Coordination, Instituto Nacional de Perinatología, Mexico City 11000, Mexico; courml@hotmail.com (M.C.-C.); eygodinez@hotmail.com (E.G.-M.); nutricionperinatal@gmail.com (C.M.-M.); viviananegrete02@gmail.com (V.N.-M.); carlapaty90@hotmail.com (C.P.G.-L.); cruz_tolentino@yahoo.com.mx (M.T.-D.); 2 Community Interventions Research Branch, Instituto Nacional de Perinatología, Mexico City 11000, Mexico; blancasuarezrico@gmail.com; 3 Research Direction, Instituto Nacional de Perinatología, Mexico City 11000, Mexico; gpestrad@gmail.com

**Keywords:** serum 25-OH-D, vitamin D status, pregnancy, rural, urban, Hispanic, Mexico

## Abstract

Background: During pregnancy, vitamin D requirements are higher due to fetal growth and development. Vitamin D production occurs mainly through sunlight exposure, which is affected by geographic location and lifestyle factors. Methods: This was a case-control study nested within two cohorts of adult pregnant women (*n* = 298): urban (Mexico City) and rural (Cuetzalan). To reduce confounding, pairs were selected by age, pregestational body mass index, and pregnancy trimester. Generalized linear models were used to assess the two groups according to their vitamin D status. Results: A total of 298 adult women were studied: 149 from a rural area and 149 from an urban area. Vitamin D deficiency and insufficiency were observed in 28% and 38.2% of women, respectively. A trend for higher 25(OH)D concentrations was observed in women from the rural area (27.5 ng/mL vs. 25.8 ng/mL), probably related to the type of job, where women with partial jobs showing less probability of having vitamin D deficiency (OR = 0.26; CI = 0.06–1.16; *p* = 0.08) and vitamin D insufficiency (OR = 0.24; CI = 0.06–0.99; *p* = 0.05). Women whose Last Menstrual Period occurred in spring showed lower vitamin D concentration compared to those whose LMP occurred in winter (*p* < 0.01). Conclusions: A high prevalence of vitamin D deficiency was observed in both rural and urban areas. Women living in rural areas tended to have higher 25(OH)D concentrations, probably related to more sunlight exposure associated with their type of job.

## 1. Introduction

Vitamin D is a liposoluble vitamin and hormone that is synthesized in the skin, and it is normally related to bone health [1]. Vitamin D is commonly known as the sun vitamin. Estimations indicate that sunlight exposure contributes to 90% of vitamin D production through the conversion of 7-dehydrocholesterol in the skin to pre-vitamin D, which isomerizes to cholecalciferol (Vitamin D_3_) [2]. Exposure to Ultraviolet B (UVB) radiation (wavelengths from 290 nm to 315 nm) stimulate the conversion process. Thus, factors such as the geographic location (latitude), the season, time of the day, pollution, and even cloudiness influence vitamin D production [2,3]. Besides the geographical situation, other factors such as skin pigmentation, diet, health status, obesity, clothing, use of sunscreen, and physical activity play a major role in vitamin D synthesis in the skin [2]. The type of mobility of pregnant women in daily life influences sunlight exposure and, thus, the vitamin D concentration. Day-to-day mobility within a space of social coexistence is determined by territorial and socioeconomic characteristics [4].

Food intake barely contributes to the body requirements of vitamin D since only a few foods contain this nutrient and they are not widely consumed [5]. Main sources coming from food in the form of vitamin D_3_ are codfish liver oil, salmon, beef liver, egg yolk, cheese, and fortified foods (margarines and some dairy). To a lesser extent, mushrooms contain the D_2_ form (ergocalciferol) of the vitamin [6]. In some countries, most of the vitamin D is obtained through nutritional supplements [6].

Previously, the relevance of vitamin D was focused on the contribution to calcium-phosphate homeostasis, which promotes healthy growth and reduces the risk of bone fractures [5,6]. Nonetheless, vitamin D has been recently implicated in cellular and neuromuscular growth, immunological modulation, tissue inflammation reduction, and tumor suppression [6].

Vitamin D and calcium participate in bone mineralization, bone accretion, and growth during the development of the fetus, therefore increasing the requirement of these nutrients during pregnancy. The dietary intake recommendation for vitamin D during pregnancy according to the Institute of Medicine of the National Academies (Food and Nutrition Board) is 600 IU/day (15 mcg/day) [7] The Royal College of Obstetricians and Gynaecologists (RCOG) recommend 400 IU/day (10 mcg/day) for all pregnant women and 1000 IU/day (25 mcg/day) for high risk women [8]. In Mexico, the dietary intake recommendation for pregnant women is 200 IU/day [9].

The major circulating form of vitamin D is 25-hydroxyvitamin D (calcidiol, 25(OH)D), and its measurement is clinically used for the assessment of vitamin D status. Even though the cut-off points for vitamin D deficiency (VD-D) and insufficiency (VD-I) have not been well established, most experts agree that an adequate vitamin D status may be defined as 25(OH)D ≥ 30 ng/mL, and a deficient state is usually considered as 25(OH)D < 20 ng/mL [10]. Globally, it has been reported that 54% of pregnant women have a VD-D [11]. VD-D during pregnancy is associated with a higher risk of preeclampsia, gestational diabetes, preterm birth, low birth weight, among other complications [12,13]. In Mexico, a cross-sectional study in mother-child binomials showed a prevalence of VD-D in 61% of pregnant women during the third trimester and in 98% of babies [14]. A recent report from a cohort of Mexican healthy pregnant women showed that only 39% of them had adequate 25(OH)D concentrations during the third trimester of gestation, and 20% had a deficient status [15].

The location and socioeconomic factors (activity, type of job, type of transportation) of pregnant women could lead to differences in sunlight exposure. Living in an urban or rural (countryside) area may affect 25(OH)D concentrations. Studies comparing maternal vitamin D status between women living in rural areas and women in urban areas are scarce. Some studies done in Vietnam and in Pakistan have reported higher 25(OH)D concentrations in women from rural areas compared with women from urban areas [16,17]). In a study comparing pregnant women from an urban zone and rural zone in Mongolia and women in Boston, women living in rural provinces of Mongolia showed higher season-adjusted 25(OH)D concentrations than their counterparts living in the capital (urban areas) [18]. No studies in women from Latin America or of Hispanic origin have evaluated vitamin D status by location or other socioeconomic factors.

The aim of the present study was to compare the vitamin D status between women living in rural areas and women living in urban areas, and evaluate the effect of other sociodemographic factors. 

## 2. Materials and Methods

This study derives from two different cohorts of pregnant women with singleton pregnancies. The design was a case-control study nested within two cohorts. Two groups were studied: one from an urban area (Mexico City) and another from a rural area (Cuetzalan, Puebla). The inclusion criteria were healthy adult women and singleton pregnancy. The authors declare that all the investigations were conducted according to the guidelines of the Declaration of Helsinki of 1975.

### 2.1. Women from an Urban Area—Mexico City

Women from this group were selected from the prospective cohort OBESO (for its Spanish acronym “*Origen Bioquímico y Epigenético del Sobrepeso y la Obesidad*”) Biochemical and Epigenetic Origin of Overweight and Obesity) carried out in the Instituto Nacional de Perinatología (INPer) Mexico City, Mexico. The study was approved in March 2017 by the INPer Ethics and Research Committee (Registry No. 3300-11402-01-575-17). The women that volunteered to participate signed an informed consent letter. All women were recruited from January 2017 to January 2020 during the first trimester of pregnancy at the Fetal Maternal Medicine Department. Only adult women with a singleton pregnancy were included. Every woman received regular prenatal care in the INPer facilities, without any modification to the usual clinical interventions. Assessments were performed during the second (18 to 22.6 weeks) and third (28 to 34.6 weeks) trimesters in the Nutrition Unit. Gestational age was calculated in every visit according to the fetal ultrasound screening at the first trimester. Height and weight were measured using a steady digital stadiometer (model 264, SECA, Hamburg, Germany) and bioimpedance equipment (model 230, Inbody, Seoul, Korea), respectively, in order to calculate the pregestational body mass index (pBMI). Women were classified as normal weight (pBMI > 18.5 < 24.9), overweight (pBMI ≥ 25), or obese (pBMI ≥ 30), according to the WHO parameters [15]. Demographic information (education, occupation, and socioeconomic status) and clinical data (parity) were obtained. Maternal blood sample acquisition was recorded considering two categories: spring/summer and autumn/winter seasons. The socioeconomic status was classified as high/medium or low, according to the INPer parameters. Women were categorized as nulliparous (with no born child) or multiparous (at least one born child).

Any micronutrient supplementation prescribed by an obstetrician-gynecologist or healthcare professional was independent of the present study. Dosage of prescribed vitamin D3 (IU/d) was calculated for each trimester according to the use of vitamin D supplements or any other multivitamin that could provide vitamin D. A previous analysis of this cohort showed that 89% of women received vitamin D supplementation throughout pregnancy or at some point in pregnancy; 50% of them received 500 IU/day.

### 2.2. Women from a Rural Area—Cuetzalan, Puebla, Mexico

Indigenous pregnant women from this group derive from the study: “*Aplicación y evaluación del modelo sociocultural para prevenir muertes maternas, en Cuetzalan del Progreso, Puebla*”, which was approved by the INPer Ethics and Research Committee in August 2017 (Registry No. 2017-1-55). All women belong to the Cuetzalan del Progreso municipality located in the Northern mountain range of Puebla, Mexico. The women that arrived between March and June 2018 at a rural clinic or medical unit in six out of the eight Town Councils that constitute the municipality were invited to participate in the latter study. The women that volunteered to participate received a verbal and written description of the study details and signed an informed consent letter afterward. 

For the data acquisition of the indigenous women, there was an approach to each clinic at the Town Councils and to the Cuetzalan General Hospital. A socioeconomic survey was applied to each woman. This instrument has been used before in the same population. Height and weight were measured using a digital floor scale (model 803, SECA, Hamburg, Germany) and a portable stadiometer (model 213, SECA, Hamburg, Germany), respectively. Pregestational weight was inquired, and the pBMI was calculated; pBMI classification was performed according to the WHO parameters (same procedure previously described). All pregnant women were supposed to receive a multivitamin with 200 IU/d of vitamin D as part of their prenatal care. Individual supplemented vitamin D doses were not recorded. A fasting blood sample was obtained from each woman and later centrifuged to obtain the serum. Serum samples were properly labeled and frozen at −70 °C in the Cuetzalan General Hospital. Transfer of the samples to the Institute was performed in controlled-temperature containers.

For this study, adolescent women were excluded. All adult women that participated in the Cuetzalan study were included.

### 2.3. Subset Selection

To avoid skewing caused by confounding factors and to achieve more homologous groups, pairs were selected from the urban study to match all women in the rural study. The following criteria were used to select women from the urban group: (1) Age: women with a maximum age difference of two years were selected from each group; (2) pBMI diagnosis: women in the same pBMI category (normal, overweight, or obese) were selected from each group, considering a maximum difference of two units in the raw pBMI value; (3) Trimester: Women within the same period assessment, whether second or third trimester, were selected from each group. Pairs were selected in a consecutive manner, using the number from the study ID.

### 2.4. Vitamin D (25-OH-D) Analysis

Sample analysis was carried out in the Nutrition and Bioprogramming Department of the Institute. Serum concentrations of 25(OH)D were analyzed via ELISA (chemiluminescence) (Architect, Abbott, Longford, Ireland). The 25(OH)D calibration curve should be run in duplicate of 6 points. Range of calibration values: 0.0 ng/mL–160.0 ng/mL using a 4 parameter logistic curve fit data calculation method to generate the calibration curve. A single replicate of each of the different concentration controls must be run to assess the assay calibration. An acceptable coefficient of variation was considered as <5%. An insufficient status was considered when serum concentrations were <30 ng/mL, and a deficient status was considered when concentrations were <20 ng/mL [10]. 

### 2.5. Statistical Analysis

The 25(OH)D concentrations were assessed using graphics and simple association patterns with variables of interest. These association patterns were evaluated using a Student *t*-test, chi-square, linear regression models, and multinomial, depending on the response variable. According to the simple association patterns, some variables were collapsed into secondary categories. For the marital status variable, the categories were married, free union, and single or divorced. Categories of the occupation variable were a full-time job, partial job, student, and housewife. We defined a full-time job and partial job as following: (1) a full-time job as a paid job in agreement with the workday journey within a fixed schedule and a specific space (secretary, warehouse assistant, nurse, teacher, among others.); (2) partial job as any activity related to an informal job (street food sale, handcraft sale). Finally, for the education variable, the categories were: no education or primary (elementary and middle school), incomplete secondary (truncated high school), complete secondary (concluded high school), and higher education (Bachelor’s degree).

The date of the last menstrual period (LMP) was recorded for all women to assess the amount of time of sunlight exposure before pregnancy and at an early stage of pregnancy. This was established due to significant differences in the serum concentration of vitamin D according to the season of the year in which the blood samples were acquired. Based on the latter, the LMP reported by the pregnant women was considered as the date of the beginning of pregnancy and therefore classified as the following: winter season, from 21 December to 19 March; spring season, from 20 March to 19 June; summer season, from 20 June to 21 September; and autumn season, from 22 September to 20 December. Using multinomial logistic regression models, the sociodemographic characteristics of pregnant women with a higher probability of being situated in the categories of VD-D or VD-I were evaluated and compared to the probability of having vitamin D sufficiency. Both the variables with a significance level *p* < 0.01 in the simple association patterns, as well as the variables with solid evidence in the literature related to 25(OH)D concentration, were included in the model. Statistical analyses were carried out using the statistical program STATA (v. 12).

## 3. Results

The final sample of the rural study was 149 adult pregnant women, so 149 pairs from the urban study were selected. A total of 298 women were studied. 

Sociodemographic characteristics of the participants are shown and arranged by location in Table 1. Women from the rural area were younger and had a lesser level of education compared to the urban counterpart, and a higher proportion had a partial job (*p* < 0.01). Moreover, significant differences were found in the reported season of LMP, where very few women in the rural group had their LMP in spring (*p* < 0.01). There were no differences in the pBMI nor in parity by location (*p* > 0.05).

**Figure 1:**
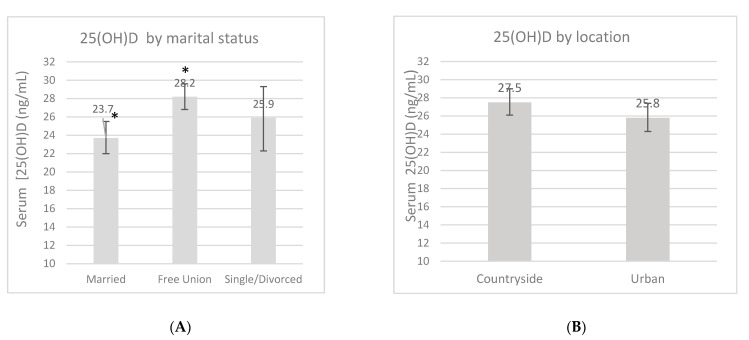

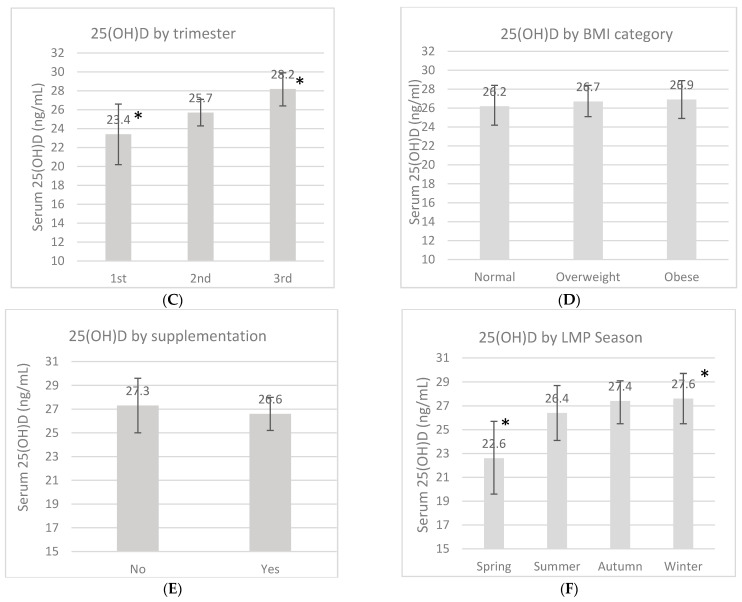
Serum 25-OH-D concentrations (ng/mL) by marital status (**A**), location (**B**), pregnancy trimester (**C**), pregestational BMI category (**D**), supplementation (**E**) and by last menstrual period (LMP) (**F**). Unadjusted linear regression. * *p* < 0.05.

An overall 25(OH)D blood measurement was recorded in 23 (7.8%) women in the first trimester, in 136 (45.6%) women in the second trimester, and in 139 (46.6%) women in the third trimester. The mean 25(OH)D concentration during pregnancy was 26.7 ± 9.4 ng/mL, and this fluctuated according to certain characteristics (Figure 1). These measurements had no difference according to the location, but a trend of higher 25(OH)D concentrations was found in women from the rural area compared to women in the urban area (27.5 ng/mL vs. 25.8 ng/mL, *p* = 0.13). There was a higher 25(OH)D concentration in the third trimester compared to the first one (*p* = 0.02). Women whose LMP occurred during spring showed lower concentrations compared to the women whose LMP occurred during winter (*p* < 0.01). When the women were categorized by marital status, those who lived in a domestic partnership had higher 25(OH)D concentrations compared to married women (*p* < 0.01). Neither pBMI nor vitamin D supplementation during pregnancy had an impact on concentrations (*p* > 0.05).

A total of 102 (34.2%) women showed adequate 25(OH)D concentrations (≥30 ng/mL). In contrast, 196 (65.8%) women showed VD-D or VD-I. A trend for higher frequency of VD-D was observed the urban group (*n* = 105) compared with the rural group (*n* = 91) (X^2^: 2.92, *p* = 0.08). Adequate status was observed in 38.8% of women from the rural area and in 29.5% of women in the urban area. All women in the rural study were prescribed a multivitamin with 200 IU/d. In the urban study, 89% of women had a supplement recommendation during pregnancy; the median dose prescribed in this study was 500 IU/d. Table 2 shows the prevalence of VD-D and VD-I according to clinical and sociodemographic factors (Table 2).

As shown in Table 3, having a full-time job was associated with a trend of a lower probability of presenting VD-D compared to being a housewife (OR = 0.26; 95%CI = 0.06–1.16; *p* = 0.08). This same effect was observed between the third and first trimesters of pregnancy (OR = 0.21; 95%CI = 0.04–1.11; *p* = 0.07). In the case of women whose LMP occurred during spring (OR= 5.7; 95%CI = 1.53–21.07; *p* < 0.001) and during summer (OR = 3.3; 95%CI = 1.01–10.84; *p* = 0.04), there was a five- and two-fold increase, respectively, in the probability of having VD-D compared to women whose LMP occurred during winter. Factors associated with a reduced probability of VD-I included having a partial job compared to being a housewife (OR = 0.24; 95%CI = 0.06–0.99; *p* = 0.05) and being in the third trimester of pregnancy compared to being in the first one (OR = 0.22; 95%CI = 0.05–1.01; *p* = 0.05).

## 4. Discussion

The present study demonstrates that a high prevalence of vitamin D deficiency (25(OH)D < 20 ng/mL) and insufficiency (25(OH)D < 30 ng/mL) was found in Mexican pregnant women from both urban (VD-D: 37.6%) and rural areas (VD-D: 29.5%). 

VD-D during pregnancy represents a worldwide health issue, which has been widely reported. Studies in developing countries manifest a prevalence of VD-D ranging from 51.3% to 100% [19]. In women from the American continent, a prevalence between 42% and 72% of vitamin D during pregnancy has been reported [11]. A cross-sectional study carried out in Mexico showed that 61% of pregnant women had VD-D during the third trimester, a higher proportion than reported in our study [14]. A previous report from the OBESO cohort showed that in pregnant women receiving prenatal care, the prevalence of VD-D in the first trimester was 37%, decreasing to 20% in the third trimester, mainly related to increased supplementation during pregnancy [10]. So, even though women from both areas were exposed to supplementation, probably the doses were not enough to achieve an adequate vitamin D status. Not even the higher doses than women from the urban group took (50% of them received 500 IU/d) were sufficient to prevent a deficient status. This is very relevant considering that VD-D has been associated with a significantly higher risk of developing GDM, preeclampsia, preterm birth, and delivering a small-for-gestational-age newborn [12,13]. According to our study, it is possible that women in urban areas have higher risks of developing adverse perinatal outcomes. On the other hand, dietary intake recommendations in Mexico and in other countries appear to be very low (200 IU/d), reducing the probability of pregnant women receiving higher doses of vitamin D supplementation.

It is worth noting that even though there were no significant differences in vitamin D concentration between the groups of urban and pregnant women in the rural area, we found a tendency to a higher concentration of vitamin D in the countryside women compared to the urban counterpart. A likely explanation could be the type of daily mobility linked to the occupation of each group of women according to the location. Interestingly, a “sort of protection” against VD-D and VD-I was observed in women that have a partial job, meaning that most of the activities are carried out in the street (food or handcraft sale). Countryside women showed a significantly greater proportion of having partial jobs, and they were probably prone to higher sunlight exposure as a consequence. Partial jobs, as a means of daily mobility, are not limited to the movement within an area, but it forms as such to allow the development of a lifestyle and day-to-day solutions that are culturally and socially codified [20,21,22]. In this matter, daily mobility for urban women is usually done through public transportation, and the most common activities are going to work, carrying children to and from school, and attending to the places of medical service [23]. In pregnant women from rural areas, daily mobility involves having to walk to either sell their goods, to wash clothes riverside, to deliver food at the husband’s workspace, or to go to the places for medical service [24], and as such, this group may be more exposed to sunlight.

In both groups of women, daily mobility entails a certain degree of sunlight exposure, although it is also influenced by geoclimatic conditions. The latitude of the locations where the studies were carried out is similar; for Mexico City, it is 19°25′ N, and for Cuetzalan it is 20°06′ N. Nonetheless, Cuetzalan has a hot semi-arid climate (18–26 °C) [25], which implies that this group of countryside pregnant women are more exposed to sunlight in comparison to women located in Mexico City, which has a temperate climate (16 °C) [26].

It is plausible to declare that the daily mobility type is crucial to understand the differences in vitamin D concentration in pregnant women that live in distinct zones of the same territory. According to the previous, three components have to be considered: day-to-day activities, space where these are performed, and the location and geoclimatic conditions. 

Women living in rural areas appear to have higher sunlight exposure and higher 25(OH)D concentrations, which may result in a lower risk for many adverse perinatal outcomes. There are no previous studies in Mexico that compare vitamin D status in pregnant women living in urban or rural zones, and there are only a few studies globally. In countries such as Vietnam, Pakistan, and Mongolia, higher 25(OH)D concentrations have been reported in pregnant women living in rural areas [16,17,18]. In a study comparing women from three different areas of Mongolia (one urban, two rural) and women from Boston, significantly lower 25(OH)D concentrations were observed in Mongolian women. However, higher concentrations were observed in women from the two rural provinces of Mongolia (15.2 and 15.3 ng/mL) compared to women in the capital (urban) (13.2 ng/mL) [18]. In another study done on non-pregnant and pregnant women in Vietnam, those living in a rural province had significantly higher 25(OH)D concentrations than women from an urban city (34 ng/mL vs. 31.2 ng/mL, respectively) [17]. 

Another important factor in Mexican women is the high prevalence of deficient intake of vitamin D. According to data from the National Nutrition and Health Survey in Mexico, 97% of non-pregnant women living in urban areas and 97% of those living in rural regions do not meet the dietary intake recommendation for vitamin D [27].

In the present study, as in many others, greater concentrations of vitamin D were observed during the third trimester compared to the first trimester. Longitudinal studies have proven that the vitamin D concentration increases during pregnancy progression [28,29,30]. There is also the possibility that women received more vitamin D supplementation later in pregnancy, considering that many women may start prenatal care very late.

Likewise, there were differences according to the season in our study. All women whose LMP occurred in spring showed the lowest vitamin D concentrations and the highest prevalence of VD-D and VD-I. This could mean that these women were in their second or third trimester of pregnancy during autumn and winter. It has been widely reported that these two seasons can reduce vitamin D concentrations due to an association with sunlight scarcity in some countries [28,29,30]. Finally, in a previous report of the OBESO cohort, in Mexican women, the 25(OH)D concentrations decreased by −1.85 ng/mL (95% CI: −2.99 to −0.72 ng/mL) in women during autumn/winter, compared to the spring/summer season [15].

One of the main strengths of this study was that we included two cultural and socially distinct populations, considering several sociodemographic variables. We were able to compare two paired groups reducing skewing and confounding factors in the associative models. We identify within the limitations that serum samples of women in the rural group were retrieved solely during spring. This forced us to analyze the seasonal effect using the LMP as a reference. An important limitation was the lack of data about vitamin D intake, including food sources and supplement doses. The cross-sectional design is another weakness.

## 5. Conclusions

The prevalence of vitamin D deficiency and insufficiency is high in Mexican pregnant women from both urban and rural areas, even though both groups were exposed to supplementation. A trend towards higher 25(OH)D concentrations was observed in women living in a rural area compared to women living in an urban area. Daily factors related to mobility, such as the type of job and the place where it is performed, can affect maternal vitamin D status. Perinatal health services should assess risk factors for vitamin D deficiency in pregnant women, including lifestyle and socioeconomic factors.

## Figures and Tables

**Table 1:** Pregnant women sociodemographic characteristics arranged by location.

	Total (*n* = 298)	Rural (*n* = 149)	Urban (*n* = 149)	*p*
Age	27.3 ± 5.8	24.7 ± 5.3	29.9 ± 5.1	<0.01 ^a^
Marital status				
Married	83 (27.9%)	17 (11.4%)	66 (44.3%)	<0.01 ^b^
Free union	178 (59.7%)	121 (81.2%)	57 (38.3%)	
Single/Divorced	37 (12.4%)	11 (7.4%)	26 (17.5%)	
Pregestational BMI status				
pBMI (kg/m^2^)	27.2 ± 4.0	27.2 ± 3.9	27.2 ± 4.0	0.97 ^a^
Normal ^d^	88 (29.5%)	44 (12.5%)	44 (29.5%)	0.99 ^b^
Overweight	125 (42.0%)	62 (41.6%)	63 (42.3%)	
Obese	85 (28.5%)	43 (28.9%)	42 (28.2%)	
Education				
No education / primary	17 (5.7%)	9 (6.0%)	8 (5.4%) ^+^	<0.01 ^b^
Incomplete secondary	121 (40.6%)	88 (59.1%)	33 (22.2%) ^+^	
Complete secondary	99 (33.2%)	39 (26.2%)	60 (40.3%) ^+^	
Higher ^e^	61 (20.5%)	13 (8.7%)	48 (32.2%)	
Occupation				
Housewife ^f^	193 (69.2%)	102 (68.5%)	91 (70.0%)	<0.01 ^b^
Partial job	23 (8.2%)	19 (12.7%)	4 (3.1%) ^+^	
Full-time job	52 (19%)	21 (14.1%)	32 (24.6%)	
Student	10 (3.6%)	7 (4.7%)	3 (2.3%)	
Parity				
No. of previous children	0 (1)	0 (1)	1 (1)	0.12 ^a^
None ^g^	155 (52.0%)	81 (54.4%)	74 (49.7%)	0.11 ^b^
One to two	134 (45.0%)	61 (40.9%)	73 (49.0%)	
Three or more	9 (3.0%)	7 (4.7%)	2 (1.3%)	
Season of LMP				
Spring	37 (13.5%)	3 (2.2%)	34 (24.3%) ^+^	<0.01 ^b^
Summer	76 (27.7%)	45 (33.6%)	31 (22.1%)	
Autumn	103 (37.6%)	59 (44.0%)	44 (31.5%)	
Winter ^h^	58 (21.2%)	27 (20.2%)	31 (22.1%)	

^+^*p* < 0.01; ^a^ Student’s *t*-test; ^b^ Unadjusted multinomial logistic test; ^c, d, e, f, g, h^ Reference category; reference group: urban zone.

**Table 2:** Proportion of women with vitamin D deficiency and insufficiency during pregnancy.

		VD-D		VD-I	
		%	(95% CI)	%	(95% CI)
Total		28.0	(23.0, 33.0)	38.2	(33.2, 43.2)
Marital status	Married	42.2	(32.2, 52.2)	32.5	(22.5, 42.5)
	Free union	21.0	(15.0, 27.0)	39.2	(32.2, 46.0)
	Single/Divorced	29.7	(14.7, 44.7)	45.9	(29.9, 61.9)
pBMI	Normal	34.1	(24.1, 44.1)	34.1	(24.1, 44.1)
	Overweight	25.8	(17.8, 33.8)	38.7	(30.7, 46.7)
	Obese	25.0	(16.0, 34.0)	41.7	(31.7, 52.6)
Location	Rural	25.2	(18.2, 32.2)	36.7	(28.7, 44.7)
	Urban	30.9	(23.9, 37.9)	39.6	(31.6, 47.6)
Occupation	Student	44.4	(14.4, 74.4)	22.2	NA
	Housewife	26.6	(20.6, 32.6)	37.5	(30.5, 44.5)
	Partial job	26.1	(8.0, 44.1)	26.1	(8.0, 44.1)
	Full-time job	35.8	(15.8, 55.8)	39.6	(19.6, 59.6)
No. of previous children	None	28.8	(21.8, 35.8)	34.0	(27.0, 41.0)
	One to two	28.4	(20.4, 36.4)	43.3	(35.5, 51.3)
	Three or more	11.1	NA	33.3	(3.3, 63.3)
Season of LMP	Spring	45.9	(29.9, 61.9)	32.4	(17.4, 47.4)
	Summer	30.7	(20.7, 40.7)	37.7	(26.3, 48.3)
	Autumn	24.5	(16.5, 32.5)	36.3	(27.3, 45.3)
	Winter	20.7	(10.7, 30.7)	46.5	(33.5, 59.5)
Trimester	First	39.1	(19.1, 59.1)	47.8	(27.8, 67.8)
	Second	30.4	(22.4, 38.4)	39.3	(31.3, 47.3)
	Third	23.9	(16.9, 30.9)	35.1	(27.1, 43.1)
Vitamin D supplementation	Yes	27.6	(20.6, 34.6)	37.5	(29.5, 45.5)
	No	28.4	(19.4, 37.4)	39.8	(29.8, 49.8)

VD-D: Vitamin D deficiency; VD-I: Vitamin D insufficiency; 95% CI: 95% Confidence interval; pBMI: pregestational body mass index; NA: Not applicable, since the confidence interval was not possible to estimate due to the sample size in the group.

**Table 3:** Multinomial logistic regression model to evaluate the probability of vitamin D deficiency and insufficiency during pregnancy.

		OR	(95% CI)	*p*
**Vitamin D Deficiency**				
Age		1.00	(0.93, 1.08)	0.97
pBMI		1.03	(0.94, 1.14)	0.50
Marital status	Married	1.08	(0.32, 3.66)	0.89
	Free union	0.43	(0.13, 1.43)	0.17
	Single/Divorced	reference		
Location	Rural	reference		
	Urban	0.98	(0.35, 2.76)	0.97
Occupation	Housewife	reference		
	Partial job	0.26	(0.06, 1.16)	0.08
	Full-time job	0.73	(0.27, 1.98)	0.54
	Student	4.44	(0.44, 44.66)	0.21
Season of LMP	Winter	reference		
	Spring	5.99	(1.56, 22.89)	<0.01
	Summer	3.21	(0.96, 10.71)	0.05
	Autumn	2.02	(0.69, 5.85)	0.19
Trimester	First	reference		
	Second	0.46	(0.09, 2.40)	0.36
	Third	0.21	(0.04, 1.08)	0.06
Vitamin D supplementation	No	reference		
	Yes	0.73	(0.32, 1.70)	0.47
**Vitamin D Insufficiency**				
Age		1.00	(0.93, 1.08)	0.87
pBMI		1.07	(0.98, 1.17)	0.11
Marital status	Married	0.75	(0.23, 2.45)	0.64
	Free union	0.74	(0.25, 2.22)	0.59
	Single/Divorced	reference		
Location	Rural	reference		
	Urban	1.19	(0.48, 2.99)	0.71
Occupation	Housewife	reference		
	Partial job	0.24	(0.06, 0.99)	0.05
	Full-time job	1.09	(0.45, 2.64)	0.85
	Student	1.80	(0.15, 21.63)	0.64
Season of LMP	Winter	reference		
	Spring	1.58	(0.45, 5.51)	0.47
	Summer	1.89	(0.68, 5.29)	0.22
	Autumn	0.91	(0.37, 2.25)	0.84
Trimester	First	reference		
	Second	0.54	(0.12, 2.49)	0.43
	Third	0.22	(0.05, 1.03)	0.06
Vitamin D supplementation	No	reference		
	Yes	0.69	(0.32, 1.50)	0.35

Vitamin D sufficiency = reference. OR: odds ratio; 95% CI: 95% Confidence interval; pBMI: pregestational body mass index; LMP: last menstrual period.

## Data Availability

The data presented in this study are available on request from the corresponding author. The data are not publicly available because this study derives from two larger studies that are ongoing.
